# Web-Based Patient Educational Material on Osteosarcoma: Quantitative Assessment of Readability and Understandability

**DOI:** 10.2196/25005

**Published:** 2022-03-24

**Authors:** Trevor Robert Gulbrandsen, Mary Kate Skalitzky, Alan Gregory Shamrock, Burke Gao, Obada Hasan, Benjamin James Miller

**Affiliations:** 1 Department of Orthopaedics and Rehabilitation University of Iowa Hospitals and Clinics Iowa City, IA United States

**Keywords:** osteosarcoma, patient education, health literacy, web-based health information

## Abstract

**Background:**

Patients often turn to web-based resources following the diagnosis of osteosarcoma. To be fully understood by average American adults, the American Medical Association (AMA) and National Institutes of Health (NIH) recommend web-based health information to be written at a 6th grade level or lower. Previous analyses of osteosarcoma resources have not measured whether text is written such that readers can process key information (understandability) or identify available actions to take (actionability). The Patient Education Materials Assessment Tool (PEMAT) is a validated measurement of understandability and actionability.

**Objective:**

The purpose of this study was to evaluate web-based osteosarcoma resources using measures of readability, understandability, and actionability.

**Methods:**

Using the search term “osteosarcoma,” two independent Google searches were performed on March 7, 2020 (by AGS), and March 11, 2020 (by TRG). The top 50 results were collected. Websites were included if they were directed at providing patient education on osteosarcoma. Readability was quantified using validated algorithms: Flesh-Kincaid Grade Ease (FKGE), Flesch-Kincaid Grade-Level (FKGL). A higher FKGE score indicates that the material is easier to read. All other readability scores represent the US school grade level. Two independent PEMAT assessments were performed with independent scores assigned for both understandability and actionability. A PEMAT score of 70% or below is considered poorly understandable or poorly actionable. Statistical significance was defined as *P*≤.05.

**Results:**

Two searches yielded 53 unique websites, of which 37 (70%) met the inclusion criteria. The mean FKGE and FKGL scores were 40.8 (SD 13.6) and 12.0 (SD 2.4), respectively. No website scored within the acceptable NIH or AHA recommended reading level. Only 4 (11%) and 1 (3%) website met the acceptable understandability and actionability threshold. Both understandability and actionability were positively correlated with FKGE (ρ=0.55, *P*<.001; ρ=0.60, *P*<.001), but were otherwise not significantly associated with other readability scores. There were no associations between readability (*P*=.15), understandability (*P*=.20), or actionability (*P*=.31) scores and Google rank.

**Conclusions:**

Overall, web-based osteosarcoma patient educational materials scored poorly with respect to readability, understandability, and actionability. None of the web-based resources scored at the recommended reading level. Only 4 achieved the appropriate score to be considered understandable by the general public. Authors of patient resources should incorporate PEMAT and readability criteria to improve web-based resources to support patient understanding.

## Introduction

Osteosarcoma is a primary malignancy of the bone, affecting 3.4 million individuals globally each year, and is the third most common cancer in the adolescent population [1]. The current treatment for osteosarcoma consists of complete surgical resection coupled with neoadjuvant and adjuvant chemotherapy. Though the introduction of adjuvant chemotherapy in the 1970s has greatly improved survival [2], the diagnosis of osteosarcoma is a significant, life-altering event for patients. In the face of imaging, diagnostic procedures, surgical management, and possible adjuvant treatment, patients may turn to the internet for additional information on their disease and its course.

Once diagnosed with a condition that involves uncertain outcomes, such as osteosarcoma, patients often turn to the internet for additional information. In 2019, approximately 90% of US adults used the internet [3], with an estimated 72% of adults accessing the internet specifically for health information [4,5]. Health literacy is a crucial component of successful health care, with previous studies demonstrating its impact on patient understanding of surgical interventions, adherence to treatment instructions, and even surgical outcomes [6-9]. Alongside growing internet usage in the United States and greater emphasis on shared decision-making, web-based patient educational materials are increasingly recognized as a key component of disseminating health information to improve health literacy in the US population [10]. Recently, the American Medical Association (AMA) and National Institutes of Health (NIH) recommend web-based health information to be written at a 6th grade or lower reading level to be fully understood by the average adult in the United States [11-15].

Most literature assessing patient educational materials has focused on readability measures [6-10,16-21]. However, readability is dependent on the complexity of vocabulary and syntax (linguistics or word order). It provides assessments of written material and is limited in the ability to effectively assess a resource’s capacity to convey data such that readers can process and act on the presented information. This limitation has been previously recognized, and the Patient Educational Materials Assessment Tool (PEMAT) was developed to provide more versatile analysis by including two key components of health information: understandability and actionability [19-21]. Understandability is as the ability of readers to process and explain key messages, while actionability is defined as the ability of readers to identify what they can do on the basis of the information presented [21]. While past literature has investigated the readability of web-based osteosarcoma patient educational material, the understandability and actionability has not been previously investigated [18]. The purpose of this study was to use the PEMAT tool to quantify understandability and readability of web-based osteosarcoma patient educational resources, in addition to standard readability algorithms, in order to create a comprehensive analysis that assesses the average patient’s ability to read, process, and act on the presented information.

## Methods

### Education Material Identification

#### Overview

Education materials were identified using the Google search engine. Google was the search engine of choice because at the time of this study, Google searches comprised 88%-92% of the web-based search market share [16,17]. To best replicate user experience, the authors chose not to use medical or journal portals, as these resources are targeted toward medical professionals and are often not easily accessible to the public. For internal validity, two independent searches were performed on March 7, 2020 (by AGS), and March 11, 2020 (by TRG). The searches were entered to imitate real user experience.

Each reviewer recorded the top 50 websites from their independent search using the term “osteosarcoma.” A Google Trends report provides analytics data of the search rate in the United States, including how commonly a specific term is searched. Additionally, various terms can be compared. This Google Trends analysis demonstrated that the term “bone cancer” was searched 4 times more than “osteosarcoma” during the time of this study. However, search results with the term “bone cancer” produced numerous websites unrelated to osteosarcoma, including metastatic lesions, Ewing sarcoma, and chondrosarcoma. Given the specificity of this study, the authors determined it was more appropriate to narrow the search to the desired topic.

Previous analyses of click-through rates report that approximately 70% or more of “clicks” originate from the first 10 search results [22-24], with previous PEMAT studies targeting the top 10 to 50 websites [25-29]. Therefore, each reviewer recorded the top 50 websites using the term “osteosarcoma.” After consolidation and removal of the duplicates, websites not meeting inclusion criteria were excluded. Inclusion criteria were websites with the primary content consisting of educational information on osteosarcoma. Exclusion criteria were news articles, primarily audiovisual resources, personal experiences (ie, patient blogs and patient stories on hospital websites), references written for health care professionals (ie, UpToDate, Merck’s Manuals), peer-reviewed journal studies, advertisements of a product or service without patient education, articles unrelated to osteosarcoma, and articles not directed at patients as the primary consumer. For example, the initial search included websites related to canine osteosarcoma, which were subsequently eliminated.

#### Qualitative Characterization

A general tabulation of qualitative website characteristics was performed via qualitative review of the following categories: (1) discussion of operative management, (2) advertisement of a physician or group that provided the described management, (3) discussion of the general background information of the disease (anatomy, pathology, prognosis, and risk factors), (4) discussion of work-up or activities related to diagnosis or preoperative management, (5) discussion of postoperative management, and (6) discussion of complications and risks of operative management. Each website was characterized with a “yes” or “no” for each category from (1) to (6), and the characteristics were reported aggregately for across all included osteosarcoma websites. No statistical analysis was performed with the website characteristics for categories (1) to (6).

### Statistical Analysis

#### Readability

The readability of included resources was quantified using objective algorithms: Flesh-Kincaid Grade Ease (FKGE), Flesch-Kincaid Grade-Level (FKGL), Simple Measure of Gobbledygook (SMOG) grade, Coleman-Liau Index (CLI), Gunning-Fog Index (GFI), and Automated Readability Index (ARI). A higher FKGE score indicates that the material is easier to read. All other readability scores represent the US school grade level. These previously validated algorithms were accessed using readability software [25,29-34]. Copyright, references, and weblinks independent of the main text were excluded from the readability analysis. 

#### Understandability and Actionability

Understandability and actionability were assessed using the PEMAT instrument, which is validated by the Agency for Healthcare Research and Quality [19-21]. The PEMAT tool assigns independent understandability and actionability scores for each educational material on a 0%-100% scale. The PEMAT tool includes items 1-19 measurable criteria that span topics such as word choice, organization, use of numbers, content, layout or design, and visual aids. Actionability includes 7 items that assess identifiable action items in the resource, how the reader is addressed, if the reader is provided with explicit steps, tools, calculations, or charts to facilitate completion of an action item. These scores were calculated utilizing the PEMAT criteria with each present criterion receiving 1 point. The number of received points was then divided by the number of possible points and multiplied by 100 [35]. A higher score represents a higher level of understandability or actionability, respectively. The PEMAT developers have established a threshold of 70% as the minimum score required for a web-based resource to be considered actionable and understandable [21].

Understandability and actionability were scored separately for each website by 2 reviewers (MKS and TRG) [19-21,35]. As used previously by the PEMAT developers [19,21], interrater reliability was calculated using Cohen κ.

#### Search Rank Analysis

Google search ranks were averaged from 2 independently conducted searches. Spearman correlation analysis was performed to assess the correlation between the search rank and its readability, understandability, and actionability. Statistical significance was defined as *P*<.05.

## Results

### Education Material Identification

A total of 53 unique web-based materials were identified. In total, 37 (70%) websites met the inclusion criteria. In total, 11 websites were excluded as primary literature, 3 were excluded as resources directed at medical professionals, and 2 canine osteosarcoma websites were excluded.

### Qualitative Criteria

Of the 37 included resources, all (100%) included background information, and 34 (92%) discussed operative management. The majority of websites (84%) discussed workup and diagnosis, while only 22 (60%) discussed the postoperative course. Risks and complications of operative management were the least included qualitative category, present in only 20 (54%) of the included resources. A total of 10 (27%) websites included an advertisement.

### Statistical Analysis

#### Readability

**Figure 1:**
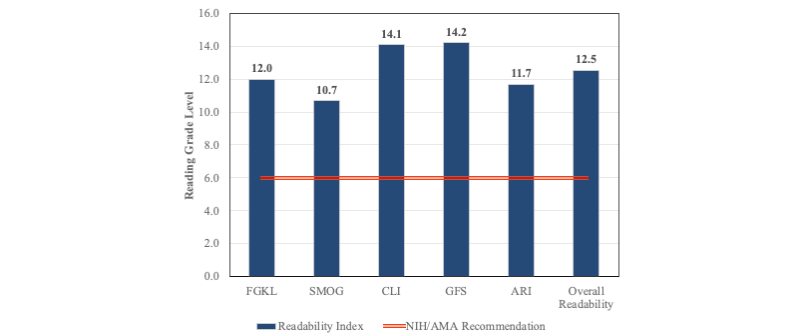
Mean readability index scores. The American Medical Association (AMA) and National Institutes of Health (NIH) recommend health information to be written at a 6th grade or lower reading level (orange line). All mean readability scores exceed this recommended reading level. ARI: Automated Readability Index, CLI: Coleman-Liau Index, FKGE: Flesh-Kincaid Grade Ease, FKGL: Flesch-Kincaid Grade-Level, GFI: Gunning-Fog Index, SMOG: Simple Measure of Gobbledygook.

The mean FKGE score was 40.8 (SD 13.6; Table 1). The mean FKGL, SMOG, CLI, GFI, and ARI scores were 12.0 (SD 2.4), 10.7 (SD 1.9), 14.1 (SD 2.0), 14.2 (SD 2.7), and 11.7 (SD 2.7), respectively. No website scores were at a 6th grade reading level or lower (Figure 1).

**Table 1:** Flesch-Kincaid Grade Ease score by website. No web-based resource was below the 8th grade reading level.

Score	School level	Interpretation	Websites, n (%)
90-100	5th grade	Easy to read and understand	0 (0)
80-90	6th grade	Easy for conversational English consumers	0 (0)
70-80	7th grade	Fairly easy to read	0 (0)
60-70	8th or 9th grade	Understood by most 13-15–year-olds	4 (11)
50-60	10th or 12th grade	Fairly difficult to read	4 (11)
30-50	College	Difficult to read	20 (54)
0-30	College graduate	Very difficult to read (University graduate level)	9 (24)

#### Understandability and Actionability

**Figure 2:**
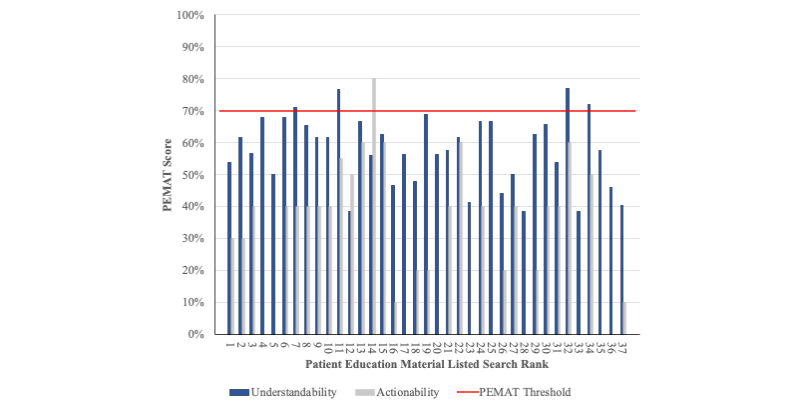
Understandability and actionability scores per website. Previous literature reports that a Patient Education Materials Assessment Tool (PEMAT) score of 70% or below is considered poorly understandable or actionable. Four patient educational resources met the understandability threshold, while only one met the actionability threshold. No resources met the threshold for both understandability and actionability.

Mean understandability and actionability scores were 57.7 (SD 10.7) and 29.1 (SD 22.6), respectively. A total of 4 (11%) and 1 (3%) website met the acceptable understandability and actionability threshold (>70%) for understandability (Figure 2). Interrater reliability demonstrated moderate agreement (Cohen κ=0.78, SD 0.003).

The criteria are listed in Table 2. The most frequently missed understandability criterion was a lack of summary (n=36, 97%), followed by lack of clear titles (n=16, 42%). While 35 (94%) scored well regarding layout and design, only 15 (39%) used visual aids, and only 15 (39%) of those specific sites had visual aids that reinforced rather than distracted from the content. Additionally, only 16 (42%) used common, everyday language and only 21 (58%) appropriately defined medical words. Both understandability and actionability were positively correlated with the FKGE score (ρ=0.55, *P*<.001; ρ=0.60, *P*<.001) but otherwise not significantly associated with other readability scores.

**Table 2:** Patient Education Materials Assessment Tool understandability and actionability scoring criteria [21].

Item				Item response		Options
**Understandability**						
	**Topic: content**					
		1	The material makes its purpose completely evident.		Disagree=0, Agree=1	
		2	The material does not include information or content that distracts from its purpose.		Disagree=0, Agree=1	
	**Topic: word choice and style**					
		3	The material uses common, everyday language.		Disagree=0, Agree=1	
		4	Medical terms are used only to familiarize audience with the terms. When used, medical terms are defined.		Disagree=0, Agree=1	
		5	The material uses the active voice.		Disagree=0, Agree=1	
	**Topic: use of numbers**					
		6	Numbers appearing in the material are clear and easy to understand.		Disagree=0, Agree=1, No numbers=N/A^a^	
		7	The material does not expect the user to perform calculations.		Disagree=0, Agree=1	
	**Topic:** **organization**					
		8	The material breaks or “chunks” information into short sections.		Disagree=0, Agree=1, Very short material^b^=N/A	
		9	The material’s sections have informative headers.		Disagree=0, Agree=1, Very short material^b^=N/A	
		10	The material presents information in a logical sequence.		Disagree=0, Agree=1	
		11	The material provides a summary.		Disagree=0, Agree=1, Very short material^b^=N/A	
	**Topic: layout and design^c^**					
		12	The material uses visual cues (eg, arrows, boxes, bullets, bold, larger font, and highlighting) to draw attention to key points.		Disagree=0, Agree=1, Video=N/A	
	**Topic:** **use of visual aids**					
		15	The material uses visual aids whenever they could make content more easily understood (eg, illustration of a healthy portion size).		Disagree=0, Agree=1	
		16	The material’s visual aids reinforce rather than distract from the content.		Disagree=0, Agree=1, No visual aids=N/A	
		17	The material’s visual aids have clear titles or captions.		Disagree=0, Agree=1, No visual aids=N/A	
		18	The material uses illustrations and photographs that are clear and uncluttered.		Disagree=0, Agree=1, No visual aids=N/A	
		19	The material uses simple tables with short and clear row and column headings.		Disagree=0, Agree=1, No tables=N/A	
**Actionability**						
		20	The material clearly identifies at least one action the user can take.		Disagree=0, Agree=1	
		21	The material addresses the user directly when describing actions.		Disagree=0, Agree=1	
		22	The material breaks down any action into manageable, explicit steps.		Disagree=0, Agree=1	
		23	The material provides a tangible tool (eg, menu planners and checklists) whenever it could help the user take action.		Disagree=0, Agree=1	
		24	The material provides simple instructions or examples of how to perform calculations.		Disagree=0, Agree=1, No calculations=N/A	
		25	The material explains how to use the charts, graphs, tables, or diagrams to take action.		Disagree=0, Agree=1, No charts, graphs, tables, or diagrams=N/A	
		26	The material uses visual aids whenever they could make it easier to act on the instructions.		Disagree=0, Agree=1	

^a^N/A: not applicable.

^b^A very short print material is defined as a material with ≤2 paragraphs and no more than 1 page in length.

^c^These items are only used for audiovisual material.

#### Search Rank Analysis

There was no association between readability (*P*=.15), understandability (*P=*.20), or actionability (*P*=.31) scores and Google rank. 

## Discussion

### Principal Findings

This study investigated the understandability, actionability, and readability of web-based resources regarding the diagnosis and treatment of osteosarcoma. Though previous literature has investigated the quality, readability, and social outreach of osteosarcoma materials [18], this is the first study to use the validated PEMAT tool to analyze patient education resources on osteosarcoma. The included osteosarcoma resources scored poorly in all readability measures. Additionally, most of these resources scored under the understandability and actionability standards with only 4 (11%) and 1 (3%) having met the acceptable threshold. Of the 53 resources included, 16 (30%) did not consist of patient education information. These findings confirm existing concerns about the lack of web-based patient material that is readily accessible and consists of high-quality content [21,36-44].

### Significance of Web-Based Patient Educational Material

Web-based patient educational material on osteosarcoma is unique, as a large proportion of the patient population consists of adolescents [1,18]. Adolescent internet usage continues to increase, with 45% of teenagers reporting near constant use of the internet, an almost doubling amount since 2015 [5]. Mass media has been cited as a health information resource for teens and is associated with changes in health behavior [45]. However, while adolescents have ready access to the internet, studies have demonstrated unique health literacy challenges within this cohort. Adolescents tend to interact less with the health care system and are therefore less familiar with its navigation [45,46]. Additionally, studies have shown that literacy is a significant challenge for adolescents, with up to 46% reading below the age correlated grade level [45-47]. Therefore, while adolescents are uniquely poised to take advantage of web-based patient resources, providers must be especially mindful of tailoring content to be readable, understandable, and actionable by this younger but technologically savvy patient population.

### Readability

Consistent with previous studies, we found osteosarcoma readability scores to be unacceptably above the NIH’s and AMA’s recommended reading level for public health content [48-50]. This study used common readability index tools and demonstrated that none (0%) of the included websites were written below a 6th grade reading level. In 2016, Cassidy et al [51] reviewed 17 readability studies consisting of orthopedic web-based patient information. They demonstrated that only 0% to 14% had appropriate readability rates using a 6th grade threshold, and only 18% were of the 8th grade reading level [51]. Lam et al [18] found similar results with osteosarcoma educational material and reported that 86% of included websites were written above the 6th-8th grade level [18]. However, rather than using the PEMAT scoring algorithm, they evaluated the qualitative aspects of osteosarcoma websites with the DISCERN instrument [51]. DISCERN criteria score quality on the basis of 16 general questions focused on the patient’s opinion of the written material [52]. Overall, DISCERN instrument is used to determine the completeness of the content but does not focus on the reader’s ability to understand or act on the material. This study used PEMAT, a validated 24-point scoring system that uses specific variables to evaluate the understandability and actionability of written and visual content.

### PEMAT

While readability instruments measure the complexity of the vocabulary and syntax, they do not directly measure the understandability and actionability. Using the reliable and valid PEMAT instrument in this study demonstrated that only 4 (11%) and 1 (3%) included osteosarcoma material met the threshold for understandability and actionability [21]. Additionally, no material met both the understandability and actionability threshold. These scores correspond to those reported in other medical and surgical subspecialities and demonstrate the lack of adequate demonstration of patient education materials on the internet [25,29-31,43,53]. Additionally, there was no association between Google rank and readability, understandability, or actionability; therefore, patients must also be made aware that top ranked websites are not necessarily equivalent with utility or quality.

The osteosarcoma resources were graded using the PEMAT criteria, websites that failed to adhere to the understandability and actionability criteria scored below the PEMAT threshold. In this study, there were several commonly missed understandability criteria across the osteosarcoma websites. The main criteria included missing summaries, lack of visual aids, and unclear titles. Frequently missed actionability criteria included failing to address the patient directly, failing to break down instructions into explicit steps, and failing to provide a tangible action tool such as a checklist. These criteria are valuable aspects of educational material as they optimize the ability for patients to adequately understand content as well as undergo simplistic actions. Missing factors inhibit patient education and can further place the patient at risk for misunderstanding vital material regarding osteosarcoma diagnosis, tests, and treatment modalities [6-9].

To adequately address these deficits, website authors should consider incorporating PEMAT guidelines to ensure the development of patient-appropriate resources [35]. For example, PEMAT guidelines recommend that materials utilize common, everyday language such as “pain killer” rather than “analgesic” [35]. By referencing PEMAT guidelines during the writing process, website authors can create web-based resources that are understandable and actionable.

### Limitations

There are limitations of this study that are important to discuss. The top 50 search results are subject to the influence of temporal changes and vary at various times and search locations. The authors cleared all cookies and cache prior to the search to mitigate some variability. The choice of search engine, search term, and country of origin can influence the search results. However, the authors utilized the most common search engine with the most specific term: “osteosarcoma.” The readability measures can be skewed by certain health care vocabulary. Words including “osteosarcoma” can inherently increase the grade level of the content. Therefore, this aspect may inflate all the grading scores used in this study. However, readability is known to have its limitation in all health care and medical content [54]. Additionally, the subjectivity of the PEMAT grading including implicit bias could not be fully eliminated. To limit this bias and subjectivity, two authors independently performed the grading, which demonstrated agreement with interrater reliability consistent with prior studies utilizing PEMAT [19].

### Conclusions

Web-based patient educational material on osteosarcoma scored poorly with respect to readability, understandability, and actionability. None of the web-based resources scored by the AMA and NIH recommended reading level, and only 4 scored above the threshold for what is considered understandable to the general public. Optimization of the most accessible osteosarcoma websites is necessary. Authors of patient resources should incorporate PEMAT and readability criteria to improve web-based resources to support patient understanding.

## Abbreviations

AMA: American Medical Association
ARI: Automated Readability Index
CLI: Coleman-Liau Index
FKGE: Flesh-Kincaid Grade Ease
FKGL: Flesch-Kincaid Grade-Level
GFI: Gunning-Fog Index
NIH: National Institutes of Health
PEMAT: Patient Education Materials Assessment Tool
SMOG: Simple Measure of Gobbledygook
